# Stuttering Speech

**Published:** 1888-10

**Authors:** 


### STUTTERING SPEECH.

Having recently attempted to cure a case of stuttering in a child, I have searched through all the homœopathic literature at my command to find if any cases of this trouble had ever been reported as cured by internal medication. No such case could be found; therefore, I now report such notes on the therapeutics of the disease, as I have been able to gather from the *Materia Medica*, and to ask if any of our readers have had any experience in treating this disease. I have greatly improved a case by the use of Mercurius, but have not cured it. The remedy was indicated by the catarrhal symptoms rather than by the stuttering. The following symptoms have been gathered from the *Materia Medica:*

Aconite.—Speech stammering; no power of articulation; he uttered only unintelligible sounds; he lost all power of speech.

Atropinum.—Frequent stuttering, especially at words difficul to pronounce; never used to do so; articluation indistinct, rapid, and chattering.

Belladonna.—Speech rapid, interrupted; speech low, impeded; stammering speech; stammers like one intoxicated; indistinct speech, stuttering; difficult speech, difficult breathing, and great lassitude; afterward anxiety; paralytic weakness of the organs of speech.

Bovista.—Stammering; he stammered at times, particularly when reading; was not able to pronounce several words.

Bufo.—Stammering; difficult, impeded, and unintelligible speech.

Cannabis-ind.—Stammering and stuttering; his lips failed of utterance as if paralyzed. (The Arsenic patient cannot speak because he cannot close his lips.)

DULCAMARA.—Stammering from time to time, as if intoxicated; indistinct articulation, though he tried constantly to speak.

EUPHRASIA.—While speaking, he recommences many times, not only repeating the first words of the sentence (a kind of stammering), but also after the periods, he frequently recommences, in order to select another expression; formerly he used to speak connectedly.

MERCURIUS.—SPEECH DIFFICULT ON ACCOUNT OF THE TREMBLING OF THE MOUTH AND TONGUE. Speech stammering and unusually very difficult; could scarcely speak from the state of agitation they were thrown in, the moment they were addressed or attempted to articulate; stammering, slow speech, difficult; entirely unable to speak on any excitement; stammered like a child; dreadful stammering; utterance embarrassed, indistinct and hurried.

OPIUM.—He is unable to talk with open mouth; he answered in a stammering manner, with interrupted articulation.

PHOSPHORUS.—Stuttered when endeavoring to articulate; speech, difficult, weak, and slow.

PLUMBUM.—Articulation imperfect, often even incomplete; sometimes, on attempting to speak he uttered only confused sounds, more or less intelligible.

SECALE.—Stammers unintelligible words between the teeth; speech, difficult and stammering; speech, slow and weak, with a feeling on every motion as if there were always some resistance to be overcome.

SELENIUM.—A kind of stammering speech, so that he made mistakes in talking, uttered syllables wrongly, and could not articulate many words at all for many days.

STRAMONIUM.—Stammering speech; difficult and unintelligible; a kind of paralysis of the organs of speech; he has to exert himself a long time before he is able to utter a word; he merely stammers and utters unconnected sounds. Can articulate, but the words he utters sound loud and harsh.

TABACUM.—While reading he cannot articulate; he reads very indistinctly, quite contrary to habit; speech difficult and unintelligible.

VIPERA.—Speech difficult and inarticulate; stammers a few unintelligible words, with weakness and sleepiness.

The patient I am now trying to cure is a strong, healthy boy, of six years. When trying to speak, the tongue is turned upward, much saliva gathers in mouth, and he keeps moving one foot or hand. Eats hastily. What is the remedy?

